# Potential antihypertensive activity of novel peptides from green basil leaves

**DOI:** 10.1186/s12906-023-04098-2

**Published:** 2023-08-08

**Authors:** Pattaneeya Prangthip, Watanalai Panbangred, Onrapak Reamtong

**Affiliations:** 1 Department of Tropical Nutrition and Food Science, Faculty of Tropical Medicine, Mahidol University, Bangkok, Thailand; 2 Research, Innovation and Partnerships Office, King Mongkut’s University of Technology Thonburi, Bangkok, Thailand; 3 Department of Molecular Tropical Medicine and Genetics, Faculty of Tropical Medicine, Mahidol University, Bangkok, Thailand

**Keywords:** Antihypertensive, Hypertension, Peptide,*Ocimum tenuiflorum*, Basil leaves

## Abstract

Hypertension is among the risk factors of death globally. Novel antihypertensive peptides are alternative choices of antihypertensive assistance. This study aimed to discover novel antihypertensive peptides from green basil leaves. Two bioactive peptides with high angiotensin-converting enzyme inhibition (Asp-Leu-Ser-Ser-Ala-Pro; peptide 1) and antioxidant (Asp-Ser-Val-Ser-Ala-Ser-Pro; peptide 2) activities were gavaged to male Wistar rats induced with *N*^*G*^-nitro-l-arginine methyl-ester (L-NAME). L-NAME-treated rats (HT) had decreased body weights and levels of nitrite and nitrate, which are metabolites of nitric oxide. The levels of their glucose and liver function indicators increased as compared to normal rats. HT rats receiving antihypertensive drugs (HTD) showed higher low-density lipoprotein and low-density lipoprotein/high-density lipoprotein levels than HT rats. Peptide 1 seems to benefit the rat lipid profiles, liver functions, antioxidant, nitrite, nitrate, and angiotensin II peptide levels but not peptide 2. In conclusion, our findings indicate the antihypertensive potential related to vasodilation of peptides from green basil leaves.

**Graphical Abstract:**

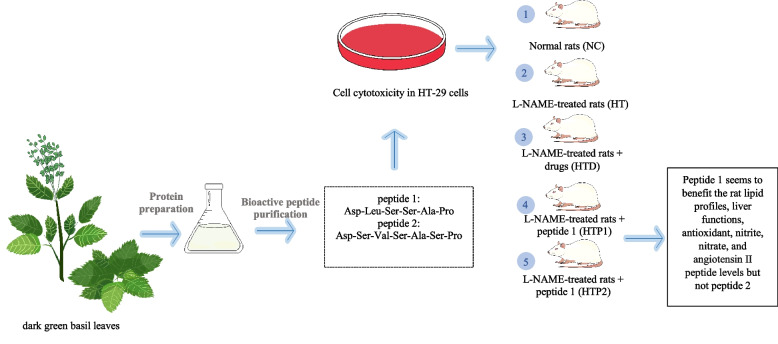

**Supplementary Information:**

The online version contains supplementary material available at 10.1186/s12906-023-04098-2.

## Introduction

Hypertension is among the risk factors of heart attack, stroke, heart failure, and other diseases, which are leading causes of death globally [1]. Angiotensin-converting enzyme (ACE) was considered to play a role in blood pressure (BP) regulation by converting inactive angiotensin I (AGE I) to active angiotensin II (AGE II), which is a potent vasoconstrictor [2]. To reduce BP, synthetic ACE inhibitors have been used as the primary approach in antihypertensive agents. Lisinopril, an antihypertensive drug approved by US Food and Drug Administration for the management of hypertension [3], is an ACE inhibitor that prevents the conversion of AGE I to AGE II. Many other drugs are available for BP control. However, side effects, such as cough, taste disturbance, skin rash, and kidney failure, were reported in some patients [4]. Novel molecules may be the alternative choices for treating hypertension and its complication. Bioactive peptides are specific protein fragments that could modulate the body’s physiological functions and exert health beneficial properties and thus are useful for the development of nutraceuticals or functional foods [5].

Several inhibitory peptides that target angiotensin I-converting enzyme (ACE) have been isolated from plant protein digests, such as spinach [6], pea [7] and sweet potato [8] due to the high efficiency of protein synthesis pathway which enables them to produce large quantities of proteins [9]. The plant genus *Ocimum*. (Lamiaceae), commonly known as basil or tulsi. The solvent extracts derived from basil leaves were found to contain substantial quantities of phenolic and flavonoid compounds, including rutin, epicatechin, vanillic acid, quercetin, caffeic acid, linalool and methyl chavico [10, 11]. Thus, there are extensively studied for its therapeutic potential. Numerous in vitro and in vivo studies have demonstrated potent pharmacological actions related on hypertension. In vitro studies demonstrated that the leaf extract possessed a range of biological activities, such as antioxidant, antimicrobial, and anticancer effects [11–14]. Animal studies also revealed that the hydro-ethanolic extract of Holy Basil leaves significantly increased antioxidant activity and decreased the level of lipid peroxidation (LPO), while also modulating immunomodulatory and anti-inflammatory effects. [12, 14–16]. Given these compelling findings, the present investigation was undertaken to assess and isolate protein digests from basil leave.

The current investigation has resulted in the isolation of two newly identified bioactive peptides with potent anti-angiotensin I-converting enzyme (ACE) inhibitory activity and antioxidant properties from green basil leaves. These peptides demonstrated no adverse cytotoxic effects on the HT-29 colon cancer cell line, even at high concentrations up to 1000 µM. Moreover, the hypotensive efficacy of these peptides was assessed in male Wistar rats with induced hypertension through the use of *N*^*G*^-nitro-l-arginine methyl-ester (L-NAME) in comparison to several other candidate molecules.

## Material and methods

### Bioactive peptide preparation

Altogether, dark green basil leaves (*Ocimum tenuiflorum* Lamiaceae from Chom Thong district, Bangkok with the latitude of 13.685896 and the longitude of 100.467758.) were prepared following the previous description [6, 8] with some modifications. Briefly, 100 g of basil leaves were washed and mixed with 100 mL of Tris–HCl acid buffer at pH level of 8.3. The mixture was blended at high speed using a blender for 1 min and centrifuged at 5,500 rcf for 30 min. Four volumes of acetone were added to one volume of supernatant obtained (200–300 mL), mixed together and stirred at 4 °C on a magnetic stirrer for 12 h or overnight. The precipitated proteins were collected by centrifugation at 5,500 rcf for 30 min, and acetone was removed using a rotary evaporator before freeze drying. Five grams of protein were further digested by Pepsin (Sigma-Aldrich Co., St. Louis, MO, USA) at a final concentration of 0.4% (w/v) with pH of 2 at 37 °C for 2 h before adjusting the pH level to 7.5 using 6 M NaOH. Then, trypsin (Sigma-Aldrich Co.) was added to obtain the final concentration of 0.4% (w/v) and incubated at 37 °C again for 2 h. The reaction was stopped by heating at 95 °C for 10 min. The supernatant obtained after centrifugation at 5,500 rcf with temperature of 4 °C for 15 min was kept at 4 $^\circ{\rm C}$ for further step of ultrafiltration using an Amicon ultra (Millipore, USA) membrane (diameter of 7.6 cm), followed by gel suspension of Sephadex G-25 (Wako Pure Chemical Industry, Japan) and eluted with deionized water at a flow rate of 1.0 mL min^−1^. The peptides concentration is assayed by a modified Lowry protein assay [17]. Peptide fractions were collected, lyophilized, and stored at − 20 °C for further analysis.

### Determination of antihypertensive and antioxidant activities

#### ACE inhibitory activity

ACE inhibitory activity assay kit (Dojindo Molecular Technology Inc., Kumamoto, Japan) was used for the measurement of ACE inhibition in proteins derived from basil leaves before and after digestion according to the company manual. Briefly, 10 µl of each sample, substrate buffer, and enzyme mixture were mixed and incubated in 96-well plate at 37 °C for 1 h before measuring ACE inhibition at 450 nm of absorption (A). The ACE inhibitory activity was calculated and calculated as follows: inhibitory activity (%) = [(A_without sample_ − A_with sample_)/(A_without sample_ − A_blank_)] × 100.

#### 1,1-Diphenyl-2-picrylhydrazyl (DPPH) radical scavenging activity

The sample (50 µl) was mixed with 0.1-mM DPPH (Sigma-Aldrich Co.) at a ratio of 1:1 as the previous description [10] with some modifications. The mixture was incubated in the dark at room temperature for 30 min before measuring an absorption (A) at 517 nm using butylated hydroxyanisole (BHA) as the standard antioxidant. Scavenging activity (%) was calculated as follows: [(A_control_ − A_sample_)/A_control_] × 100.

#### 2,2’-azinobis (3-ethylbenzothiazoline-6-sulphonic-acid) (ABTS) radical scavenging activity

ABTS stock solution consists of 7-mM ABTS and 2.45-mM potassium persulfate (Sigma-Aldrich Co.) and kept in dark at room temperature overnight before use. The stock solution was diluted with distilled water to make an ABTS working solution with an absorbance of 0.70 $\pm$ 0.02 at 734 nm. The sample (10 µl) was mixed with 200 μl of ABTS working solution. The absorbance was measured at 734 nm after incubating in the dark at room temperature for 5 min [18].

#### Oxygen radical absorbance capacity (ORAC)

ORAC was performed as the following modifications [19]. Briefly, 25 µL of samples or standards (Trolox, Sigma-Aldrich Co) was added to 150 µL of the 1X fluorescein solution (Sigma-Aldrich Co.) in a 96-well microtiter. After incubating the plate for 30 min at 37 °C, 25 µL of free radical initiator solution (Sigma-Aldrich Co.) was add into each well. The absorbance was then measured with a fluorescent microplate reader at 37ºC at excitation/emission of 480/520 nm. The area under the curve was calculated between 1 and 5 min for a total of 60 min.

Gel filtration chromatography and reverse-phase high performance liquid chromatography (RP-HPLC).

Peptide fractions with a molecular mass of < 1 kDa was purified by gel filtration chromatography (Sephadex G-25) twice using different gel lengths of 1.5*30 and 1.5*70 cm for the 1^st^ and 2^nd^ gel columns, respectively. The protein sample of 500 mg was resuspended with 250 µl of distilled water and vortex to obtain a homogenous suspension. The mixtures were centrifuged at 1,400 rcf at 4 $^\circ{\rm C}$ for 5 min. The supernatants ($\sim$ 1 mL) were loaded onto SP Sephadex G-25 equilibrated and eluted with deionized water at a flow rate of 1.0 mL min^−1^. Each fraction was kept at − 20 °C. The active fraction was subjected to RP-HPLC on XTerra (Waters Corperation, Milford, MA, USA) RP18 column, 5 µm and 4.6 × 250 mm with a linear gradient of acetonitrile (5%–30% in 35 min) containing 0.1% trifluoroacetic acid at a flow rate of 0.3 mL/min. The eluted peaks were detected at 210 nm. The fraction from RP-HPLC chromatogram was collected and assayed for anti-ACE and antioxidant [18]. The fractions of peptides that showed bioactivities, such as antioxidative and/or ACE inhibition, were collected and pooled from 10 injections, lyophilized, and stored at − 20 °C. The fractions with the highest activities were further analyzed by mass spectrometry for their molecular mass and amino acid sequences.

#### Bioactive peptide identification and sequence analysis using mass spectrometry

Peptide fractions from purified peak of RP-HPLC were analyzed by liquid chromatography with tandem mass spectrometry (LC–MS/MS) on MicroToF Q II (Bruker Daltonics, Germany) to predict their amino acid sequences and molecular mass. To perform this analysis, the peptides were acidified with 0.1% (v/v) formic acid and injected an Ultimate 3000 capillary LC system (Dionex, Camberley, UK). The separation was performed under a flow rate of 300 nL min^−1^. The pressure of the column and loading were 90 and 58 bars, respectively. The eluent was sprayed using a capillary voltage of 35 kV into a nanoelectrospray source of Q-ToF. The tandem mass spectra cover the mass range of m/z 50–1500. The LC–MS/MS data were analyzed using DataAnalysis version 3.4 (Bruker Daltonics, Bremen, Germany). The scores of ions from MS/MS were calculated to protein scores using Mascot software (Matrix Science Inc., Boston, MA, USA). Protein scores are the sum of the highest ion score for each sequence, and the correction was calculated by the total number of molecular mass match [18].

#### In silico prediction of ACE inhibitor peptides

The three-dimensional structure of human ACE was imported from the Protein Data Bank (PDB: 1O8A). The ligand or peptide structure was generated from Gaussview 3.09 (Gaussian Inc., Pittsburgh, PA, USA). Autodock Tools, Cygwin terminal, and Ligplot plus were used to study the ability of bioactive peptides to bind with ACE at the active site. If a ligand can bind to an active site of the enzyme, the binding energy shows a negative value [18]. Peptides showing negative binding energy values were selected for further synthesis to determine their ACE inhibitory activity.

#### Synthetic peptides

Synthetic active peptides were ordered from GenScript (Piscataway, NJ, USA) using a solid-phase peptide synthesis technique and stored at − 20 °C until use.

#### Determination of cell cytotoxicity

The HT-29 cell line (5 × 10^4^ cells/mL) were seeded into 24-well plate and incubated in Dulbecco’s modified eagle medium supplemented with 10% fetal bovine serum (FBS), 1% penicillin/streptomycin solution, 1% pyruvate, and 1% non-essential amino acid at 37 °C and 5% carbon dioxide (CO_2_) for 24 h. The cells were treated with peptides from dark green basil (100 µl) at concentrations of 0.1, 1, 10, 100, and 1000 µM overnight. After washing the cell once with 1X Phosphate-buffered saline (PBS), 0.5-mg/mL 3-(4,5-dimethylthiazol-2-yl)-2,5-diphenyltetrazolium bromide (MTT) solution (500 µl/well) was added and incubated at 37 °C in the dark for 30 min. The MTT solution was removed and 300 µl of acidified isopropanol (0.01% 6-M HCl) was added. Then, 150 µl from each well was transferred into 96-well plates and the absorbance was measured at OD570 and OD630. Cell viability (%) was calculated as follows: [(A_570_–A_630_)_treatment_/(A_570_–A_630_)_Media_] × 100 [20].

#### Animal experiment

Male Wistar rats from Nomura Siam International (Bangkok, Thailand) aged 5 weeks were used following the rules and regulations of the Animal Care Ethical Committee of Laboratory Animal Science Center, Faculty of Tropical Medicine, Mahidol University (approval of certificate no: FTM-AUC 004/2021). The experiments were also carried out in adherence to the Ethical Principles and Guidelines for the Use of Animals National Research Council of Thailand, American Veterinary Medical Association Guidelines for the Euthanasia of Animals: 2013 edition and Canadian Council on animal care. The experiments are reported in accordance with ARRIVE guidelines 2.0. PS Power and sample size calculation program [http://powerandsamplesize.com/Calculators/Compare-2-Means/2-Sample-1-Sided] was chosen to calculate the sample size using previous data [21]. 5 rats per group is enough for this study to show the difference in the hypertensive rats and normal rats. After allowing to accustom in a new environment for a week, two to three rats were then housed per cage with a solid bottom and open top in a light–dark (12:12) controlled room with an automatic ambient humidity (65% ± 10%) and temperature of 25 °C  ± 2 °C; they were allowed free access to standard chow diet and water until the end of experiment.

#### L-NAME and lisinopril preparation

L-NAME and lisinopril were ordered from Merck Millipore Thailand. L-NAME was diluted in distilled water to obtain the final concentration of 40 mg/1 mL and immediately gavaged to rat at a dose of 40 mg/kg body weight (BW)/day [21]. Lisinopril was diluted in distilled water to obtain the final concentration of 10 mg/1 mL and immediately orally gavaged to rat the dose of 10 mg/kg BW/day [22].

#### Rat experimental design

After 1 week of acclimation, 25 male Wistar rats were separated randomly into two groups of control rat (NC; *n* = 5) with distilled water and L-NAME-treated rat (*n* = 20). At this step, distilled water was orally gavaged to NC rat at 1 mL/100 g BW. L-NAME at a final concentration of 40 mg/kg BW in 1 mL distilled water was orally gavage to induce hypertension in rat at a dose of 40 mg/kg BW for 3 weeks. Thereafter, L-NAME treated rats were randomly classified into the following four groups: L-NAME untreated rat (HT; *n* = 5); L-NAME + lisinopril rat (HTD; *n* = 5); L-NAME + peptide 1 at 30 mg/kg rat (HTP1; *n* = 5); and L-NAME + peptide 2 at 30 mg/kg rat (HTP2; *n* = 5); for the remaining 3 weeks. NC (*n* = 5) was still orally gavaged with distilled water. The peptides were diluted in distilled water to obtain a final concentration of 30 mg/1 mL and immediately gavaged to rat at a dose of 30 mg/kg BW/day). Lisinopril was diluted in distilled water to obtain a final concentration of 10 mg/1 mL and immediately orally gavaged to rat at a dose of 10 mg/kg BW/day. At the end of the experiment, each rat was fasted for approximately 12–16 h and euthanized with CO_2_ inhalation. Thereafter, the whole blood of each rat was drawn from the inferior vena cava, taken in a non-heparinized tube, and immediately centrifuged at 1,400 rcf at 4 °C for 15 min. The separated serum was collected and preserved at − 80 °C until further analysis. Then, the liver, heart, spleen, and kidneys were collected, trimmed, washed with normal saline, wiped with filter paper, and weighed.

#### Lipid profile, glucose, kidney and liver function, and ORAC levels

One thousand microlite of serum was used for measuring the lipid profiles, including total cholesterol, triglyceride (TG), low-density lipoprotein (LDL)-cholesterol (C), high-density lipoprotein (HDL)-C, glucose levels, kidney (blood urea nitrogen and creatinine concentrations) and liver function [alanine aminotransaminase (ALT), aspartate transaminase (AST), and alkaline phosphatase (ASP)] parameters using the Cobas 6800/8800 systems (Roche Group, Switzerland). ORAC in rat serum was measured with a fluorescent microplate reader at Ex/Em = 480/520 nm, as previously mentioned.

#### Analysis of biomarkers related to hypertension

The levels of nitrite and nitrate, which are metabolites of nitric oxide (NO), were measured using 50 µl of rat serum utilizing the calorimetric method (Nitrite/nitrate Kit, Sigma-Aldrich, 2 Science Park Dr, SG), according to company protocol. Angiotensin II peptide levels were quantified by using EIA kits (Angiotensin II EIA, Sigma-Aldrich, MO, USA), as per the manufacturer’s instructions).

### Statistical analysis

Kolmogorov–Smirnov test was used to test the normality of the distribution. To determine the normal distribution of weight and blood biochemistry test data, a one-way analysis of variance was performed with a post-hoc Tukey’s multiple comparison test. The skewed distribution data of AGE II peptide were analyzed using the independent sample Kruskal–Wallis test. Differences in data values were determined using SPSS® statistics software version 18 (IBM Inc., Armonk, NY, USA). Data are considered significant at a *p*-value < 0.05.

## Results

### Antihypertensive activity of the fractions from green basil leaves

Crude extract (5.79% yield dried weight to fresh weight) were digested with pepsin at pH 2.0 and followed by trypsin at pH 7.5. Then, crude hydrolysate was fractionated into six fractions according to their molecular masses, which were as follows: < 1, 1–3, 3–5, 5–10, 10–30, and > 30 kDa. The peptide fraction with molecular mass of < 1 kDa appeared to have the lowest the concentration needed to inhibit 50% (IC50) of the ACE activity (*n* = 3) at 1.14 ± 0.03 μg/mL when compared to 1–3, 3–5, 5–10, and 10–30 kDa with mean ± standard deviation values of 1.34 ± 0.05, 1.88 ± 0.06, 1.96 ± 0.10, 4.20 ± 0.15, 4.39 ± 0.23, respectively.

### Antioxidant activity of different fractions

**Fig. 1:**
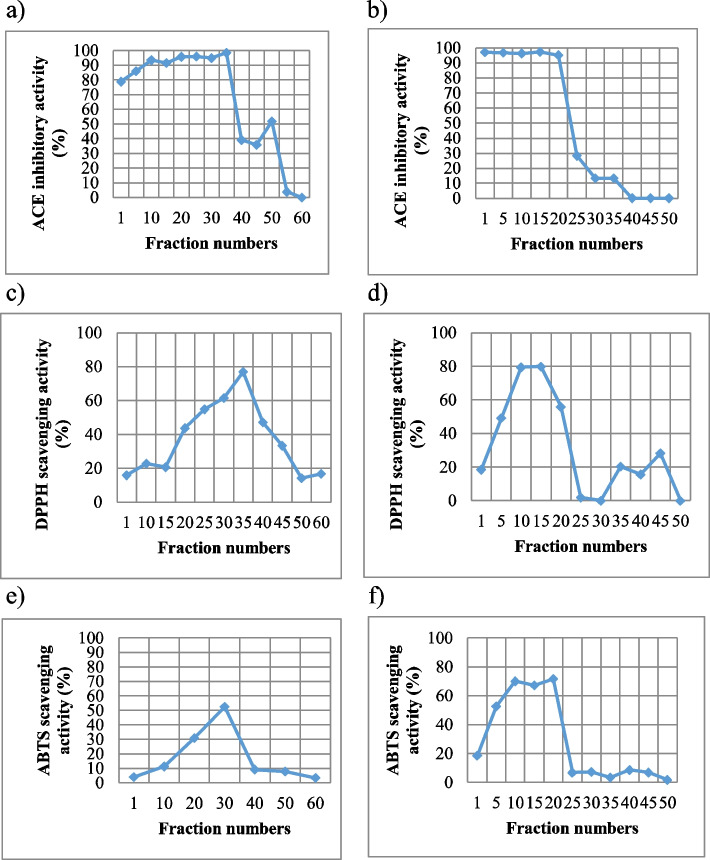
Antioxidant activities of green basil peptides with molecular mass less than 1 kDa. ACE inhibitory of the fractions were obtained from the gel column 1.5 × 30 cm (**a**) and 1.5 × 70 cm (**b**). ACE scavenging activity of the fractions were obtained from the gel column 1.5 × 30 cm (**c**) and 1.5 × 70 cm (**d**). DPPH scavenging activity of the fractions were obtained from the gel column 1.5 × 30 cm (**e**) and 1.5 × 70 cm (**f**) sequences analysis by LC–MS/MS

Compared to other fractions, the peptide fractions with a molecular mass < 1 kDa displayed the highest antioxidant activity by exhibiting the lowest IC50 of DPPH and ABTS at 1.85 ± 0.17 and 1.78 ± 0.03 μg/mL, respectively (Table 1). Peptide fractions with a molecular mass < 1 kDa were examined to determine antioxidant activities. The eluted fractions with the highest ACE inhibitory activity (95%) were found from 20 to 35 min (Fig. 1a) and 1 to 20 min (Fig. 1b) for the 1^st^ and 2^nd^ prepared peptide fractions, respectively. The eluted fractions had slightly increased DPPH radical scavenging activity at approximately 80% at 35 min (Fig. 1c) and 10–15 min (Fig. 1d) for the 1^st^ and 2^nd^ prepared peptide fraction, respectively. The ABTS profile of green basil peptides from the first gel column showed the highest radical scavenging activity of > 52% in the eluted fraction at 30 min (Fig. 1e), whereas the radical scavenging activity obtained from the 2^nd^ gel column was the highest at 71.74% in the eluted fraction between 10 and 20 min (Fig. 1f).

**Table 1:** Antioxidant scavenging activities of the fractions from green basil leaves

Peptide fraction (kDa)	IC_50_ value of antioxidant activity (μg/ml)	
	DPPH	**ABTS**
** > 30**	14.11 ± 0.07	7.60 ± 0.01
**10–30**	11.77 ± 0.07	6.23 ± 0.01
**5–10**	7.47 ± 0.18	5.36 ± 0.13
**3–5**	6.18 ± 0.15	4.74 ± 0.06
**1–3**	5.63 ± 0.31	3.64 ± 0.02
** < 1**	1.85 ± 0.17	1.78 ± 0.03
**BHA**	2.95 ± 0.20	

Values represent IC_50_ values $\pm$ SD (*n* = 3)

### RP-HPLC profile of pooled fractions and their antioxidant activities

**Fig. 2:**
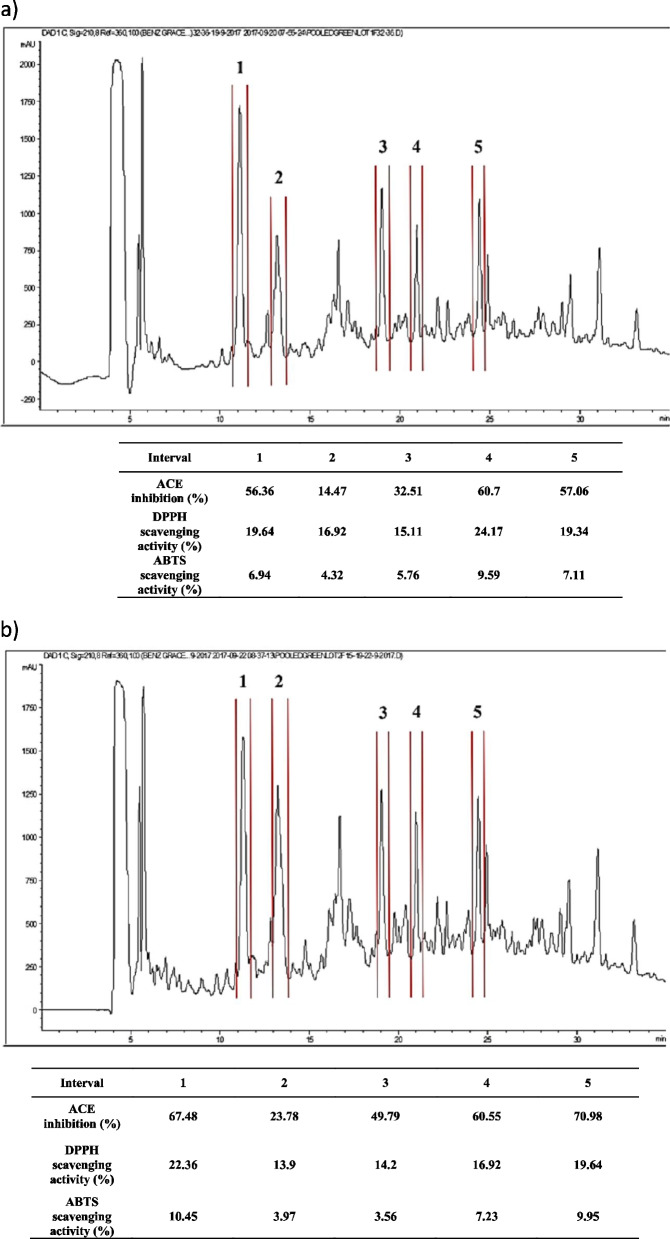
RP-HPLC profile and antioxidant activities of the green basil active peptides fraction from gel column 1.5 × 30 cm (**a**) and 1.5 × 70 cm (**b**)

The eluted fractions exhibiting both the highest antihypertensive and antioxidant activities from the same column were pooled and further purified using RP-HPLC. The RP-HPLC profile of pooled fractions from the 1^st^ (Fig. 2a) and 2^nd^ (Fig. 2b) gel columns showed many peptide peaks. The 1^st^ pool fraction was grouped into five intervals (1–5) at 10.70–11.50, 12.90–13.80, 18.70–19.40, 20.50–21.30, and 24.10–24.80 min, respectively. The 2^nd^ gel column could be grouped into five intervals (1–5) at 11.00–11.80, 13.00–13.90, 18.80–19.50, 20.70–21.30, and 24.10–24.80 min, respectively. Each interval was collected and lyophilized immediately. The peptides from each interval was dissolved with distilled water (250 µl), and measured for both anti-ACE and antioxidant activities. For the 1^st^ pool fraction, intervals 2 and 3 showed ACE inhibition ability ranging from 14.47% to 32.51%. The high ACE inhibition was found in intervals 1, 4, and 5 at 56.36%, 60.70%, and 57.06%, respectively. These three intervals also provided a high antioxidant activity for both the DPPH and ABTS assays (Fig. 2a). Therefore, intervals 1, 4 and 5, which showed a potent activity in terms of ACE inhibition and antioxidation, were purified and examined for their peptide sequences using LC–MS/MS. For the 2^nd^ pool fraction, intervals 1, 4, and 5 showed a potent activity in terms of ACE inhibition at 67.48%, 60.55%, and 70.98%, respectively. Additionally, these three intervals also provided a high antioxidant activity for both the DPPH and ABTS assays. Therefore, peptide sequence analyses were performed on these intervals.

### Molecular mass determination and antihypertensive and antioxidant peptides

The mass spectrometry analysis of the purified peptides from RP-HPLC was performed using LC–MS/MS analysis QToF (MicroToF Q II) (Bruker Daltonics, Germany) for their peptide sequence and mass prediction. Then, the characteristics of ACE inhibitory activities were examined using Autodock 1.5.6 and Cygwin terminal software [23, 24]. These programs can stimulate the interaction of peptide at the enzyme’s active sites, which are reported as binding energy values. Therefore, the binding energy value between peptides and active site of ACE can be used to predict the ACE inhibitory peptides (Table 2). Captopril was used as a positive control for this analysis since it is a common antihypertensive drug used in several studies. The positive values of binding energy implied that the peptides might not be able to bind with ACE at the active site. Therefore, the peptides with negative energy values were used as candidates for peptide synthesis and examined to confirm their antihypertensive activities (Table 2). The peptide Asp-Leu-Ser-Ser-Ala-Pro with a molecular mass of 589.283 Da showed the lowest binding energy value of − 7.13 kJ/mol and the highest activity in ACE inhibition with the lowest IC_50_ value at 4.75 µM, as compared to other synthetic peptides. The ORAC value of peptide Asp-Leu-Ser-Ser-Ala-Pro was 0.17 µmol TE/g, whereas that of Asp-Ser-Val-Ser-Ala-Ser-Pro was 0.85 µmol TE/g. The peptide Asp-Leu-Ser-Ser-Ala-Pro exhibited low antioxidant and high ACE inhibition activities, whereas the peptide Asp-Ser-Val-Ser-Ala-Ser-Pro showed low anti-ACE and high ORAC antioxidant activities was selected to compare.

**Table 2:** Molecular mass determination and amino acid sequence prediction and In silico prediction results for binding energy and hydrophobicity of peptides derived from green brasil

Sequences	Molecular mass (Da)	Binding energy with ACE (kJ/mol)	Hydro-phobicity at pH 6.8	IC_50_ (µM)
				**ACE inhibition**
Tyr-Ser-Phe	416.182	-5.6	51.67	44.23
Glu-Gly-Ser-Gly-Lys-Gly-Val-Arg	789.422	10.02	0.38	87.24
Asp-Leu-Ser-Ser-Ala-Pro	589.283	-7.13	5	4.75
Leu-Asp-Ser-Lys-His-Glu-Ala-Pro	896.448	11.03	-1.38	120.53
His-Cys-Gly-Asp-Gly-Asp-Ala-Pro-Tyr	934.337	10.17	0.56	103.05
His-Val-Leu-Ser-Ala	526.299	-5.3	44	10.02
Pro-Thr-Thr-Lys-Thr-Asn-Val-Pro-Pro-His-Phe	1238.653	203.78	1.64	119.45
Asp-Ser-Val-Ser-Ala-Ser-Pro	662.3	-3.52	0.14	117.04
Glu-Gly-Ser-Gly-Lys-Gly-Val-Arg	789.422	10.02	0.38	87.24
Captopril		-5.91	-	0.00197
BHA				ND

*ND* Not determined

### Cell cytotoxicity of synthetic peptides in HT-29 cells

The formazan crystals indicated the viability of cells, since they are produced by the reaction between enzymes from the metabolism activities of living cell and MTT. The percentage of HT-29 cell viability after peptide treatment at concentrations of 0.1–10 µM of all peptides was approximately 88%–97%. It seems that these concentrations were acceptable. The higher the concentration, the lower the cell viability (Supplementary material 1.).

BW of rats during oral administration of L-NAME and after the oral administration of the active peptide in L-NAME-treated rats.

**Fig. 3:**
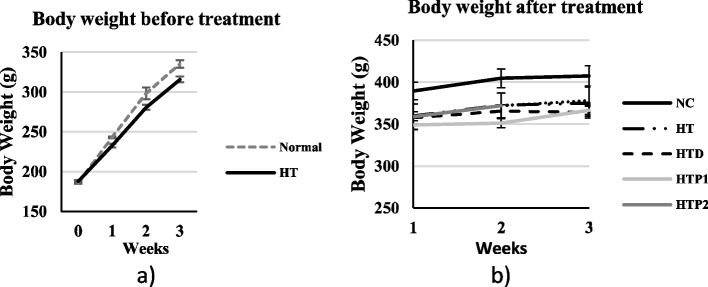
Body weight of rats orally administered L-Name for 3 weeks; Normal: rat + distilled water, HT: rat + L-Name (**a**) and continuing treatment with peptide for other 3 weeks (*n* = 5) (**b**); NC: rat + distilled water, HT: rat + L-Name, HTD: rat = L-NAME + Lisinopril, HTP1: rats + LNAME + peptide1, HTP2: rat + L-NAME + peptide2

After 3 weeks of oral administration of L-NAME, L-NAME-treated rats had lower BW than rats gavaged with distilled water (Fig. 3a). Thereafter, L-NAME-treated rats were divided into four subgroups for the remaining 3 weeks. The BW of L-NAME-treated rats was still lower than the untreated rats. Either lisinopril or peptides appeared not significantly affected the rat BWs, because no significant difference in the BWs was observed between the lisinopril- or peptide-treated and L-NAME-treated rats (Fig. 3b).

Organ weights, platelet count, and white blood cell levels after orally administering the active peptide in L-NAME-treated rats.

The weight of the livers in the L-NAME treated groups (HT, HTD, HTP1, and HTP2) was significantly lower than those of the NC group. There is no significant difference in the weights of other organs (Supplementary material 2). There is no significant difference in the platelet count and white blood cell levels between the two groups. However, the monocyte level tended to be lower in the L-NAME-treated group (HT, HTD, HTP1, and HTP2) than in the NC group. Treatment with either peptides or lisinopril did not affect the platelet count and white blood cell levels of the L-NAME treated group (Supplementary material 3.)

Blood biochemistry tests in rats after orally administering the active peptide in L-NAME-treated rats.

The increase in the glucose level of the L-NAME-treated rat (HT, HTD, HTP1, and HTP2) was higher than that of NC rats. The lipid profile levels were not different between the HT and NC rats. However, the LDL-C and LDL-C/HDL-C levels was higher in HTD rats than in NC and HT rats. Supplementation with peptides did not affect the lipid profiles of the groups. The kidney function parameters were also not different among the rat groups. For liver function, the ALP and ALT levels in seemed to be higher in the HT rats than in the NC rats, and seemed to decrease in HTD and HTP1 rats, but not in the HTP2 rats (Table 3).

**Table 3:** Lipid profiles, glucose, kidney function and livers function levels after 3 weeks treatment of peptides derived from green brasil

	NC			HT			HTD			HTP1			HTP2		
	Mean		S.E	Mean		S.E	Mean		S.E	Mean		S.E	Mean		S.E
Chol (mg/dl)	78.75	±	6.43	78.80	±	9.41	86.25	±	4.31*	75.25	±	3.50	74.75	±	5.25
Tri (mg/dl)	226.25	±	37.13	209.40	±	24.82	179.25	±	23.76	208.50	±	36.39	207.00	±	19.11
HDL (mg/dl)	32.25	±	1.80	31.80	±	2.31	32.00	±	0.82	31.50	±	1.89	31.75	±	2.10
LDL (mg/dl)	5.00	±	1.08	5.60	±	0.93*	7.00	±	0.91	5.25	±	0.63	5.25	±	0.25
VLDL (mg/dl)	45.00	±	7.44	41.80	±	5.09	35.75	±	4.70	47.75	±	9.33	47.75	±	3.79
Cho/HDL	2.43	±	0.07	2.46	±	0.15*	2.69	±	0.09	2.39	±	0.06	2.35	±	0.03
LDL/HDL	0.15	±	0.03	0.17	±	0.02	0.22	±	0.03	0.17	±	0.01	0.17	±	0.01
Glucose (mg/dl)	217.50	±	18.75*	264.80	±	20.56	263.00	±	9.68	262.25	±	13.37	235.50	±	33.82
BUN (mg/dl)	17.40	±	0.96	18.96	±	0.51	19.83	±	0.78	19.70	±	0.54	20.33	±	0.65
ALP (U/L)	142.25	±	20.08	154.60	±	13.49	129.75	±	6.66	135.00	±	3.37	159.00	±	7.43
ALT (U/L)	26.00	±	2.48	42.80	±	5.43	41.50	±	3.23	31.50	±	1.85	38.50	±	1.89
AST (U/L)	76.25	±	6.26	74.40	±	3.98	67.75	±	3.47	67.00	±	3.08	74.00	±	2.38
ORAC (µmol TE/dl)	0.25	±	0.03	0.17	±	0.02	0.19	±	0.03	0.21	±	0.01	0.27	±	0.01

^*^were considered significant at a *p*-value < 0.05

The mean and standard error of serum concentrations of nitrate, nitrite, and total nitrate and nitrite (NOx) in the NC group were 36.74 ± 7.12, 42.00 ± 7.80, and 78.74 ± 14.91 ng/µl, respectively, which are significantly higher than those of the L-NAME-treated groups (HT, HTD, HTP1, and HTP2). Only the nitrate, nitrite and NOx levels in the HTP1 group at 19.82 ± 5.51, 22.30 ± 6.03, 42.12 ± 19.99 ng/µl seemed to increase, when compared to those of the HTD group (15.05 ± 1.95, 16.17 ± 2.99, and 31.22 ± 4.94 ng/µl, respectively) (Supplementary material 4). The median (interquartile range) serum concentration of AGE II in pg/mL tended to increase in the HT group [10.16 (9.80)] as compared to that of the NC group [5.27 (0.0)]. The median AGE II level in the HTD group [5.19 (4.82)] seemed to be lower in HTP1 [5.10 (16.78)] and HTP 2 than that of the HT group [2.68 (15.32)] (Supplementary material 5).

## Discussion

Basil leaves grown in several regions worldwide contain several bioactive compounds, proteins, and amino acids [25]. This plant is considered as alternative option for bioactive peptides. This study was conducted to purify and characterize the bioactive peptides obtained from green basil leaves. Purified peptides at the dose of 20 – 50 mg/kg showed the positive effects on hypertensive rat [26–28]. The 30 mg/kg active peptides were administered to rats with induced hypertension using L-NAME in this study.

Several studies have reported on the properties of beneficial peptides for hypertension, suggesting that they should have a low molecular mass. Peptides with a molecular mass ranging from 500 to 1500 or with 2–10 amino acid residues usually showed higher ACE inhibition and antioxidant activities than those with other masses [29]. In our study, pepsin and trypsin were used to digest the green basil leave proteins, which were then fractionated and loaded onto size exclusion chromatography to separate the peptides based on their size. The peptide fractions were further purified using RP-HPLC and examined for their antioxidant and anti-ACE properties.

ACE inhibitors are medications generally indicated for hypertension. Captopril and lisinopril are the first-choice drugs for BP treatment, because the ACE inhibitor drug that is most effective remains uncertain [30]. Captopril is taken with an empty stomach. Lisinopril tends to more effective in patients compared to captopril [31]. Thus, these drugs were chosen as positive controls in the present study’s in vitro and in vivo investigations. In our study, the IC_50_ value of captopril was 1.97 nM. Our experiment showed that the peptide with a molecular mass of < 1 kDa exhibited antihypertensive activities as compared to captopril and showed an antioxidant activity as compared to peptides with other sizes. From our result, Asp-Leu-Ser-Ser-Ala-Pro showed the most potential in terms of anti-ACE and antioxidant activities in both in vitro and in vivo studies.

Several previous studies have reported that antihypertensive peptides consist of hydrophobic amino acids. The important factor in the inhibition of ACE was the tri-amino acid sequences at the C-terminal end. The amino acid residues that are preferred to be located on the second to the last position were Val, Ile, Ala, Arg, Phe, and Tyr, whereas aromatic (Tyr, Trp, Phe, and Pro) and aliphatic amino acids (Ile, Ala, Leu, and Met) are preferred to be located at the last position of the C-terminal end of peptides [32]. Most tri-peptides display a high ACE inhibition activity. The peptides containing proline at C-terminus may be resistant to degradation of gastrointestinal enzymes [33]. In our study, all predicted peptides were analyzed for their amino acids and hydrophobicity. Several previous studies reported that antioxidant peptides contain one or more hydrophobic amino acids. Glu-, Gln- and Lys-containing peptides could exhibit hydroxyl radical scavenging activities [34]. Most antioxidant peptides were reported to have moderate IC_50_ values of > 100 µM [35]. The beneficial effect of functional food on human health has been of interest for many years. Several scientists have studied on the benefit of food-derived peptides.

In our study, two similar structures of peptides (Asp-Leu-Ser-Ser-Ala-Pro, with antihypertensive potential; and Asp-Ser-Val-Ser-Ala-Ser-Pro, with a high antioxidant activity) were further investigated in rats with induced hypertension using L-NAME. Our HT rats showed no changes in blood biochemistry data. Compared to the HT rats, L-NAME-treated rats receiving antihypertensive drugs (HTD) had decreased TG and increased LDL-C and LDL-C/HDL-C levels. Previous studies suggest that abnormally low TG levels and higher ratio of non-HDL to HDL were associated with a higher risk of heart disease [36, 37]. Our HT rats also showed increased levels of liver function indicators, including ALP and ALT enzymes. An increase in the liver function indicator allows the prediction of the type of liver disease and liver tissue affected. Low TG levels is also link to impaired liver function that diminished the oxygen supply casing increased levels of inflammation [38]. HTP1 and HTP2 showed improvement in the ratio of non-HDL to HDL and ALT levels, as compared to the HTD group.

Oxidative stress is a significant contributor to the development of endothelial dysfunction, inflammation, and vascular remodeling, all of which play a role in the pathogenesis of hypertension [39]). It has been observed that individuals with hypertension commonly experience oxidative stress and dyslipidemia [40]. Our study's findings support the notion that both antihypertensive and antioxidant peptides have a positive impact on non-HDL levels. These results are consistent with a previous study that highlighted the lipid-lowering effects of bioactive substances possessing antihypertensive and antioxidant properties [41]. For example; berberine, an alkaloid derived from plants, is a natural bioactive substance that possesses both lipid- and blood pressure-lowering effects. It effectively inhibits oxidative stress, activates AMP-activated protein kinase (AMPK), and improves lipid profiles and insulin sensitivity. Berberine is recommended as a preventive measure for cardiovascular disease in individuals with high cholesterol and hypertension, with daily dosages typically ranging from 500 to 1500 mg. [42–44]. Green tea extract is other a natural bioactive substance containing polyphenols, including epigallocatechin-3-gallate (EGCG). It possesses cardioprotective and antioxidant properties, reducing lipid peroxidation and oxidized LDL levels. It also activates AMPK, inhibits HMG-CoA reductase, and interferes with cholesterol absorption [45]. Garlic-derived polysulfides, particularly S-allylcysteine, also reported to regulate endothelial NO, leading to vasodilation and blood pressure reduction [46]. Meta-analyses have confirmed the effectiveness of aged garlic extract in lowering blood pressure and improving lipid profiles [47]. Additionally, various antioxidant compounds have demonstrated antihypertensive effects by targeting distinct pathways, including the modulation of the renin-angiotensin system, vasodilation, and sodium excretion [39]. The beneficial effects of these peptides can be attributed to their ability to modulate oxidative stress, inflammation, and vascular function, thereby improving lipid profiles.

L-NAME-induced hypertension leads to the development of oxidative injury in various tissues and organs in rats [48]. The generation of reactive oxygen species is one of the major mechanisms involved in tissue damage. This may result in decreased BW (Fig. 3). However, the increased ORAC level seen in the HTP1 and HTP2 groups have showed no benefit on BW in this study.

Nitrite and nitrate are the metabolites recycled in the blood and tissue to form NO, a key vasodilator component and also known as an endothelium-derived relaxing factor [49, 50]. The lowering NO level results in the increasing BP. NO is usually measured as the sum total of nitrate and nitrite contents [51]. L-NAME-treated rats showed a significant decrease in the sum total of serum nitrite and nitrate levels. Treatment with linosinophil (HT) and peptide 2 (HTP2) alone in rats did not show any significant increase in NO levels, as compared with the NC rats. Supplementation with peptide 1 (HTP1) but not peptide 2 (HTP2) in L-NAME-treated rats tend to result in recovery of the nitrite and nitrate levels. However, compared to the HT group, the AGE II levels seemed to be lower in the HT group than in the HTP1 and HTP 2 groups. AGE II is a peptide hormone that raises the BP. Both NO and AGE II play a role in the pathogenesis of hypertension. NO is reported to antagonize the Ang II activation by downregulating the Ang II receptors ad increasing the vasodilator tone [50]. The increase in the level of both NO products and decrease in the level of AGE II in HTP1 might prove the benefit of HTP1 on hypertension, as compared to HTP2. To optimize the health of hypertensive individuals, further research is needed to understand the specific mechanisms involved and effectively target both the antihypertensive and antioxidant pathways. The major strength is in vivo confirmation. It also noted that larger sample size and longer experimental duration in this study would helpful in the interpretation of reliable results.

## Conclusion

Our results suggest that the ACE inhibition capacity of peptide Asp-Leu-Ser-Ser-Ala-Pro is important than the antioxidant activity of peptide Asp-Ser-Val-Ser-Ala-Ser-Pro in rats with induced hypertension using L-NAME, indicating that peptide 1 Asp-Leu-Ser-Ser-Ala-Pro derived from green basil leaves has a potential peptide to minimize complications or injuries from hypertension induced by L-NAME.

### Supplementary Information

**Additional file1: Supplementary material 1. **Percentage of Formazan crystal staining of HT-29 cells viability after 24 hr incubating with peptide Asp-Leu-Ser-Ser-Ala-Pro (1a) or Asp-Ser-Val-Ser-Ala-Ser-Pro (1b) at different concentrations; 0.1, 1, 10, 100 and 1000 µM and followed by incubating with 0.5 mg/ml MTT for 30 mins, and cells observed under microscope. **Supplementary material 2.** The weight of organs after 3 weeks treatment of peptides derived from green brasil. **Supplementary material 3.** Platelet counts and white blood cells levels after 3 weeks treatment of peptides derived from green brasil. **Supplementary material4.** Serum concentrations of a) nitrite, b) nitrate and c) total nitrate and nitrite (NOx). **Supplementary material 5.** Serum concentrations of serum angiotensin II peptide (AGE II) Data are represented as median (IQR). *P* = 0.352.  

## Data Availability

Data will be made available on reasonable request to the corresponding authors, Pattaneeya Prangthip.

## Abbreviations

L-NAME: *N*^*G*^-Nitro-l-arginine methyl-ester
ACE: Angiotensin-converting enzyme
BP: Blood pressure
AGE I: Angiotensin I
AGE II: Angiotensin II
DPPH: 1,1-Diphenyl-2-picrylhydrazyl
BHA: Hydroxyanisole
ABTS: 2,2’-Azinobis (3-ethylbenzothiazoline-6-sulphonic-acid)
RP-HPLC: Reverse-phase high performance liquid chromatography
FBS: Fetal bovine serum
CO_2_: Carbon dioxide
PBS: Phosphate-buffered saline
MTT: 3-(4,5-Dimethylthiazol-2-yl)-2,5-diphenyltetrazolium bromide
BW: Body Weight
TG: Triglyceride
LDL: Low-density lipoprotein
HDL: High-density lipoprotein
ALT: Alanine aminotransaminase
AST: Aspartate transaminase
ASP: Alkaline phosphatase
NO: Nitric oxide
