# Supplementary material for: Potential antihypertensive activity of novel peptides from green basil leaves
Source: BMC Complement Med Ther. 2023 Aug 8;23:282. doi: 10.1186/s12906-023-04098-2 (PMC10410819; doi:10.1186/s12906-023-04098-2)
Supplement: Supplementary file 1 — Additional file1: Supplementary material 1. Percentage of Formazan crystal staining of HT-29 cells viability after 24 hr incubating with peptide Asp-Leu-Ser-Ser-Ala-Pro (1a) or Asp-Ser-Val-Ser-Ala-Ser-Pro (1b) at different concentrations; 0.1, 1, 10, 100 and 1000 µM and followed by incubating with 0.5 mg/ml MTT for 30 mins, and cells observed under microscope. Supplementary material 2. The weight of organs after 3 weeks treatment of peptides derived from green brasil. Supplementary material 3. Platelet counts and white blood cells levels after 3 weeks treatment of peptides derived from green brasil. Supplementary material4. Serum concentrations of a) nitrite, b) nitrate and c) total nitrate and nitrite (NOx). Supplementary material 5. Serum concentrations of serum angiotensin II peptide (AGE II) Data are represented as median (IQR). P = 0.352. [file 12906_2023_4098_MOESM1_ESM.docx]

1a) 1b)

**
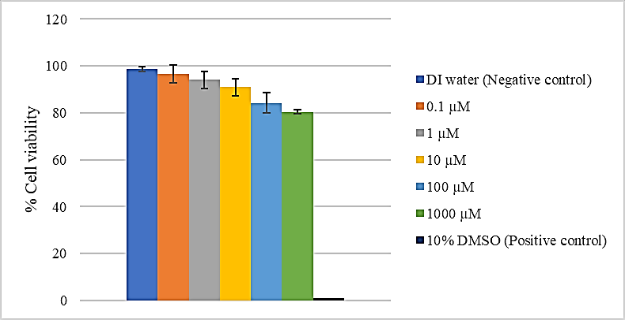
**
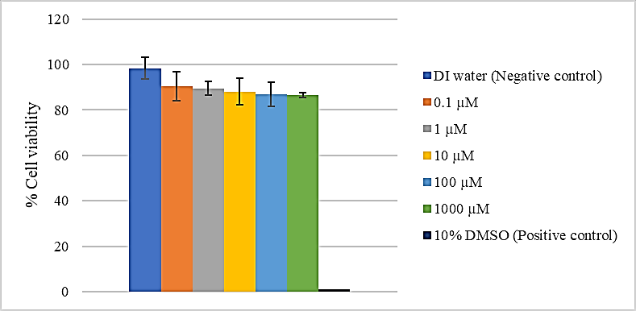


**Supplementary material 1.** Percentage of Formazan crystal staining of HT-29 cells viability after 24 hr incubating with peptide Asp-Leu-Ser-Ser-Ala-Pro (1a) or Asp-Ser-Val-Ser-Ala-Ser-Pro (1b) at different concentrations; 0.1, 1, 10, 100 and 1000 µM and followed by incubating with 0.5 mg/ml MTT for 30 mins, and cells observed under microscope.

**Supplementary material 2.** The weight of organs after 3 weeks treatment of peptides derived from green brasil

**Supplementary material 3.** Platelet counts and white blood cells levels after 3 weeks treatment of peptides derived from green brasil

# *

# *

# *

# **Supplementary material 4.** Serum concentrations of a) nitrite, b) nitrate and c) total nitrate and nitrite (NOx).

# Data are represented as mean ± SE. * were considered significant at a *p*-value < 0.05

**
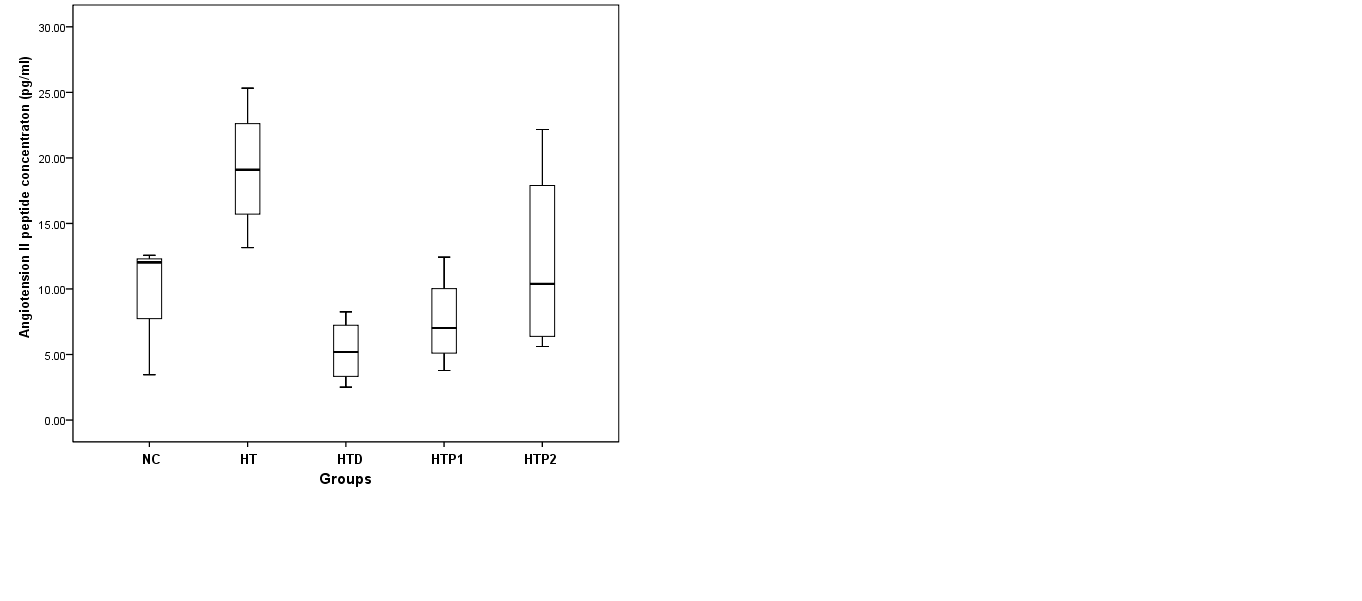
**

# **Supplementary material 5.** Serum concentrations of serum angiotensin II peptide (AGE II)

Data are represented as median (IQR). P = 0.352
